# Evaluation on quality consistency of *Ganoderma lucidum* dietary supplements collected in the United States

**DOI:** 10.1038/s41598-017-06336-3

**Published:** 2017-08-10

**Authors:** Ding-Tao Wu, Yong Deng, Ling-Xiao Chen, Jing Zhao, Anton Bzhelyansky, Shao-Ping Li

**Affiliations:** 1 State Key Laboratory of Quality Research in Chinese Medicine, Institute of Chinese Medical Sciences, University of Macau, Macao, China; 2 The United States Pharmacopeial Convention, Rockville, MD USA

## Abstract

*Ganoderma lucidum* is a well-known medicinal mushroom. At present, numerous *G*. *lucidum* products have emerged in the form of dietary supplements in the United States due to its various benefits. However, the quality consistency of these products based on their label ingredients has seldom been evaluated due to the lack of a suitable toolkit. In this study, 19 batches of products of *G*. *lucidum* (Red Reishi, Reishi), herbal/mushroom supplements purchased in the United States, were evaluated based on their bioactive components including triterpenes and polysaccharides by using chromatographic methods and saccharide mapping. The results showed that the measured ingredients of only 5 tested samples (26.3%) were in accordance with their labels, which suggested the quality consistency of *G*. *lucidum* dietary supplements in the U.S. market was poor, which should be carefully investigated.

## Introduction

Dietary supplements are consumed with intent to derive important health benefits. An estimated one hundred millions of Americans spend more than $28 billion on dietary supplements every year^1^. However, under Dietary Supplements Health and Education Act of 1994 (DSHEA), quality of dietary supplements is not evaluated by the U.S. Food and Drug Administration (FDA)^1, 2^. Both industry and FDA acknowledge that many products have been introduced into the market without any safety assessment^1^. Alarmingly, hundreds of products marketed as supplements have been found spiked with illicit substances not listed on the products’ labels, risking serious injury or even death^3^. Indeed, within the first three months of 2015, FDA warned about or recalled over 30 tainted sexual enhancement products containing prescription erectile-dysfunction drugs or related synthetic analogues^4^. In February 2015, the New York State Attorney General compelled major retailers – Walgreens, Walmart, Target, and GNC – to halt sales of certain herbal supplements, after deoxyribonucleic acid (DNA) barcoding results failed to detect DNA from the botanical materials listed on the label of ~80% tested products^5^. Though the absence of DNA might be an artifact caused by its destruction during the manufacturing process, it still triggered a great concern on the quality of dietary supplements in the United States. Usually, chemical characters of herbal supplements are more stable than their DNA sequences during different manufacturing processes. Therefore, taking chemical insights into the quality of herbal/mushroom supplements should be more reasonable.

*Ganoderma lucidum*, known as “Lingzhi” in China or “Reishi” in Japan, is a well-known medicinal mushroom and traditional Chinese medicine, which has been used for the prevention and treatment of a variety of diseases such as bronchitis, allergies, hepatitis, immunological disorders, and cancer^6, 7^. Due to its various benefits in protecting human health, numerous *G*. *lucidum* products have emerged in the form of dietary supplements in the United States, and it is also listed in the Dietary Supplements and Herbal Medicines of USP. However, their quality consistency to label ingredients has seldom been evaluated due to the lack of a suitable toolkit to audit ingredient and adulterants. Usually, triterpenes and polysaccharides are considered as the main bioactive components in *G*. *lucidum* fruiting body^7, 8^, which have been used as markers for *G*. *lucidum* officially recorded in Chinese Pharmacopoeia (2015) due to their anti-cancer and immunomodulatory activities.

In this study, the quality consistency to their label of 19 batches of *Ganoderma lucidum* (Reishi, Lingzhi) dietary supplements purchased in the United States was evaluated using a reliable and scientific toolkit including colorimetric assay, high performance thin layer chromatography (HPTLC), gas chromatography coupled with mass spectrometry (GC-MS), saccharide mapping based on polysaccharide analysis using carbohydrate gel electrophoresis (PACE), and high performance size exclusion chromatography coupled with multi angle laser light scattering and refractive index detector (HPSEC-MALLS-RID) method.

## Results and Discussion

### HPTLC fingerprints of triterpenes in *G*. *lucidum* dietary supplements

**Figure 1:**
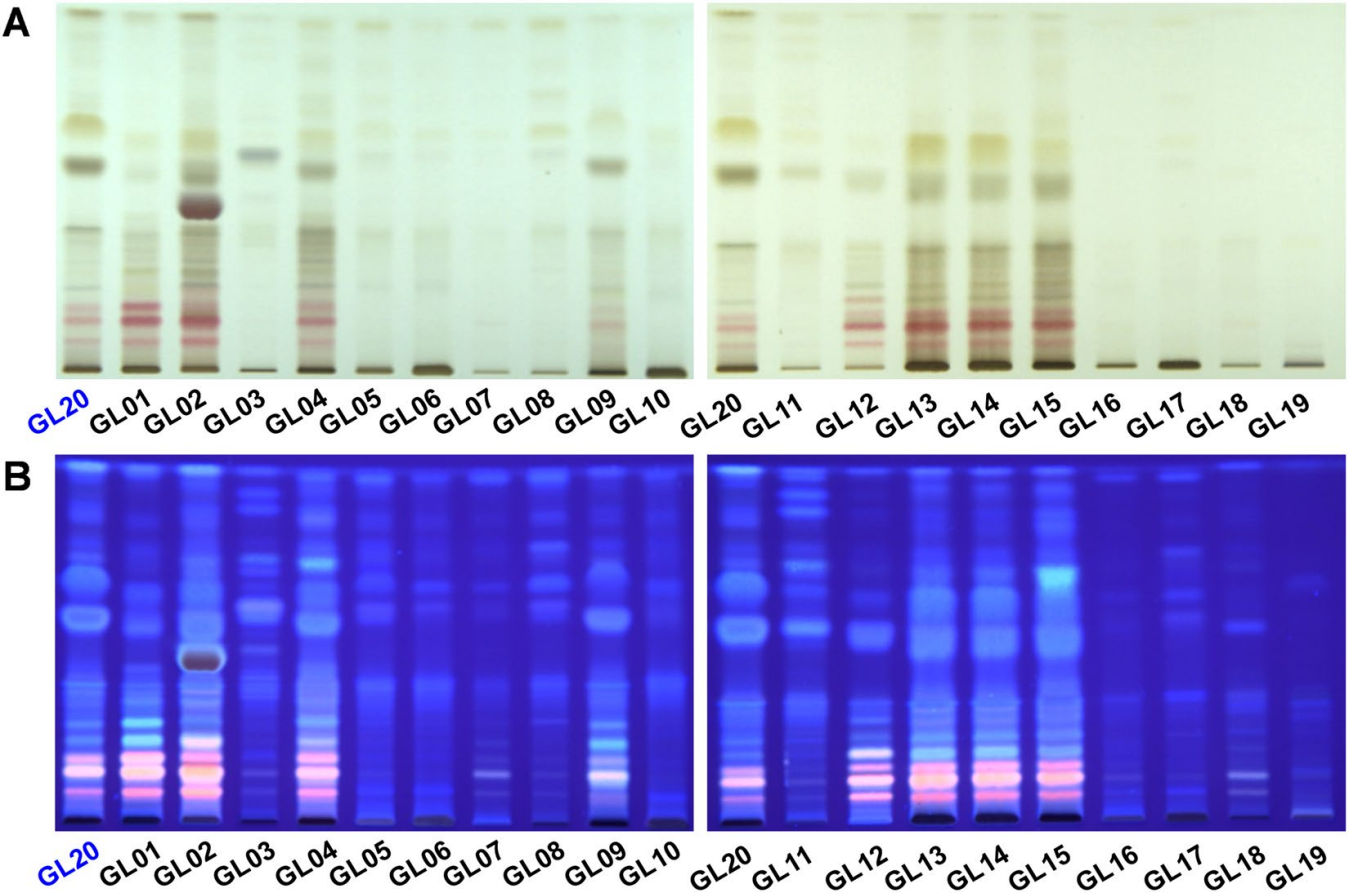
HPTLC fingerprints of triterpenes in *G*. *lucidum* dietary supplements. (**A**) Photographed under white light; (**B**) photographed under UV 365 nm; Sample codes were the same as in Table 1.

HPTLC is a simple and rapid technique for the routine quality control of herbal medicines, which provides picture-like chromatograms with special colors^9^. Indeed, HPTLC fingerprints of triterpenes in fruiting body of *G*. *lucidum* collected from different regions of China were roughly consistent, which exhibited with pink or red bands under white light after being colorized^10^. Therefore, an authenticated fruiting body of *G*. *lucidum* (GL20) was used as the reference material. Figure 1 showed the HPTLC fingerprints of ethanol extracts from *G*. *lucidum* dietary supplements under white light and ultraviolet (UV) 365 nm, respectively. The results showed that HPTLC fingerprints of triterpenes (pink/red bands in Fig. 1) in *G*. *lucidum* dietary supplements (GL01-GL19) produced by different manufacturers were remarkably different, and triterpenes closely related to *G*. *lucidum* (GL20) were only detected in 8 out of 19 (42.1%) tested products based on HPTLC analysis.

### Colorimetric assay of polysaccharides in *G*. *lucidum* dietary supplements

**Figure 2:**
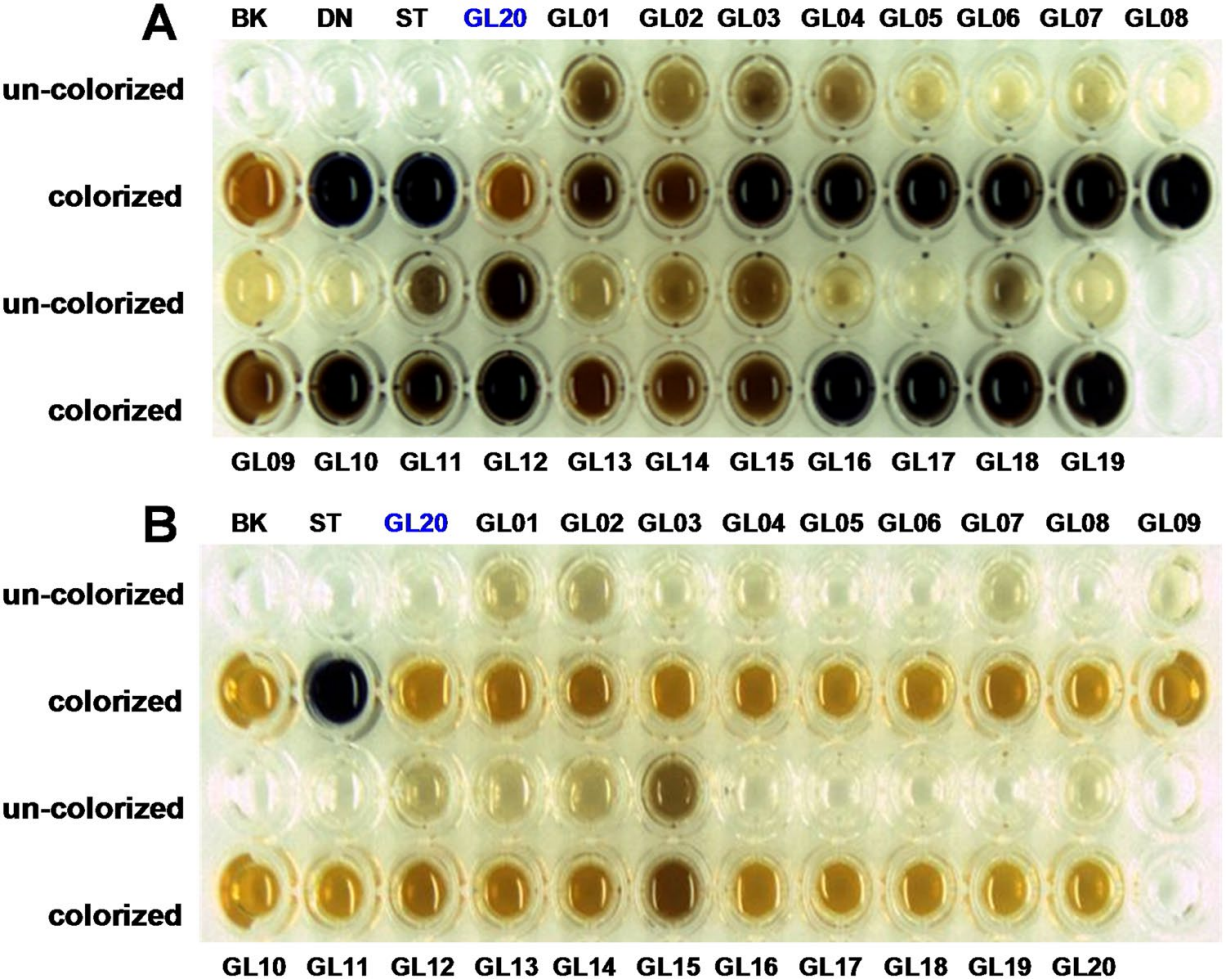
Colorimetric assay of polysaccharides in *G*. *lucidum* dietary supplements before (**A**) and after (**B**) α-amylase digestion. **BK**, water used as blank control; **DN**, malto-dextrin; **ST**, starch. Sample codes were the same as in Table 1.

Starch-like polysaccharides, absent in fruiting body of *G*. *lucidum*, are frequently employed as excipients, or neutral fillers in dietary supplements. Therefore, in order to identify the presence of starch or malto-dextrin in the dietary supplements, the colorimetric assay with iodine-potassium iodide (I_2_-KI) reagent was performed. Starch or malto-dextrin should be excluded for the determination of polysaccharides from *G*. *lucidum* dietary supplements. As shown in Fig. 2, starch-like polysaccharides were detected in 13 out of 19 (68.4%) tested products before α-amylase digestion rather than *G*. *lucidum* (GL20). Indeed, after the treatment with α-amylase, the positive response of starch-like polysaccharides in these products to I_2_-KI reagent was disappeared (Fig. 2B), which further confirmed that starch-like polysaccharides were present in *G*. *lucidum* dietary supplements.

### Saccharide mapping of polysaccharides in *G*. *lucidum* dietary supplements

**Figure 3:**
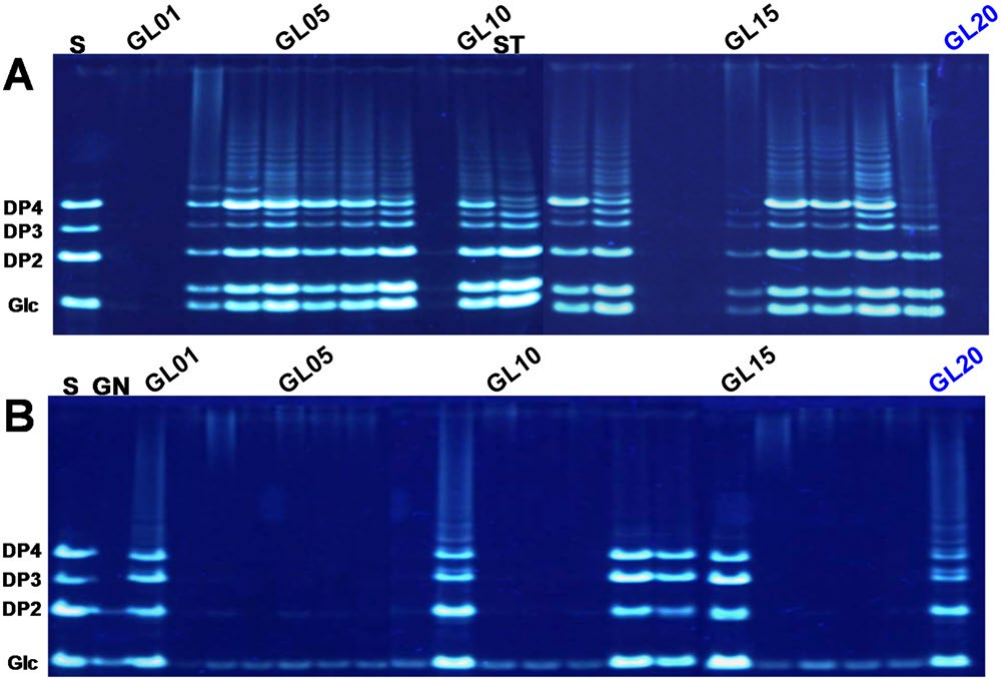
PACE fingerprints of α-amylase (**A**) and 1,3-β-D-glucanase (**B**) digested polysaccharides in *G*. *lucidum* dietary supplements. **S**, mixture of glucose (**Glc**), laminaribiose (**DP2**), laminaritriose (**DP3**), and laminaritetraose (**DP4**); **S1**, mixture of glucose (**Glc**), galactose (**Gal**), laminaribiose (**DP2**), laminaritetraose (**DP4**), and D-galacturonic acid (**GalA**); **ST**, enzymatic digestions of starch; GN, enzymatic digestions of β-1,3-glucan; The sample codes were the same as in Table 1.

**Figure 4:**
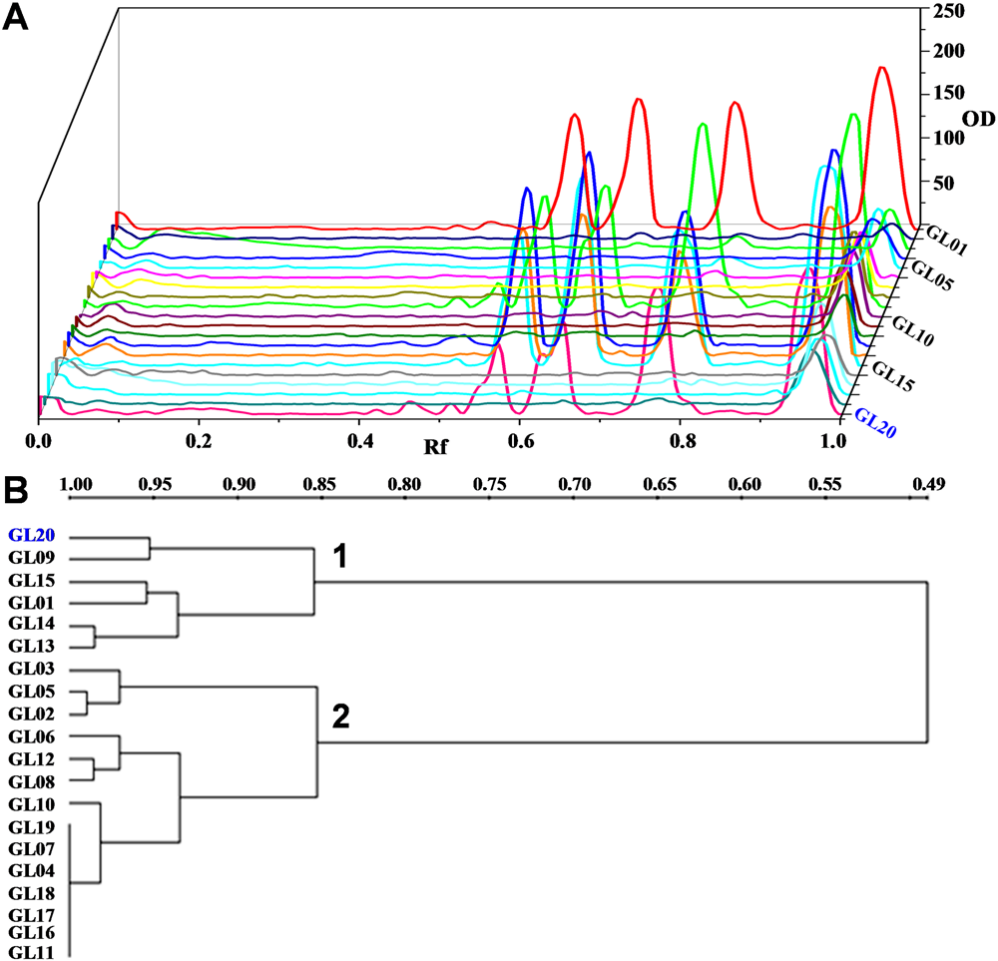
The digital scanning chromatograms (**A**) for PACE fingerprints of 1,3-β-D-glucanase digested polysaccharides and their dendrograms of hierarchical clustering analysis (**B**). Sample codes were the same as in Table 1.

Generally, the major bioactive polysaccharide in *G*. *lucidum* is known as branched 1,3-β-D-glucan^6, 7, 11^, a specific polysaccharide with immunostimulatory and anti-tumor activities. Indeed, PACE fingerprints of 1,3-β-D-glucanase digested fingerprints of polysaccharides in *G*. *lucidum* collected from different regions of China were similar. Saccharide mapping based on PACE analysis also had advantages of high resolution and high sensitivity^12^. Therefore, 1,3-β-D-glucanase and α-amylase were selected for enzymatic digestion of polysaccharides from *G*. *lucidum* dietary supplements, then the enzymatic hydrolysates were analyzed by using saccharide mapping based on PACE analysis. Figure 3A showed the PACE fingerprints of α-amylase digested polysaccharides from *G*. *lucidum* dietary supplements. The results showed that the positive response on α-amylase was found in most tested samples, which further confirmed that starch-like polysaccharides existed in *G*. *lucidum* dietary supplements. Furthermore, Fig. 3B showed the PACE fingerprints of 1,3-β-D-glucanase digested polysaccharides in *G*. *lucidum* dietary supplements, which indicated that PACE fingerprints of 1,3-β-D-glucanase digested polysaccharides from GL01, GL09, GL13, GL14 and GL15 were greatly similar to that of *G*. *lucidum* (GL20). However, other tested samples were obviously different from that of GL20. Hierarchical cluster analysis was further performed based on the digital scanning chromatograms of PACE fingerprints of 1,3-β-D-glucanase digested polysaccharides (Fig. 4). As shown in Fig. 4B, dietary supplements including GL01, GL09, GL13, GL14, and GL15, together with GL20 could be clustered into the same group (group 1), which confirmed that polysaccharides in these samples were greatly similar. The data indicated that branched 1,3-β-D-glucan, a specific polysaccharide with immunostimulatory and anti-tumor activities in *G*. *lucidum*, was only detected in 5 out 19 (26.3%) tested products.

### GC-MS fingerprints of polysaccharides in *G*. *lucidum* dietary supplements

**Figure 5:**
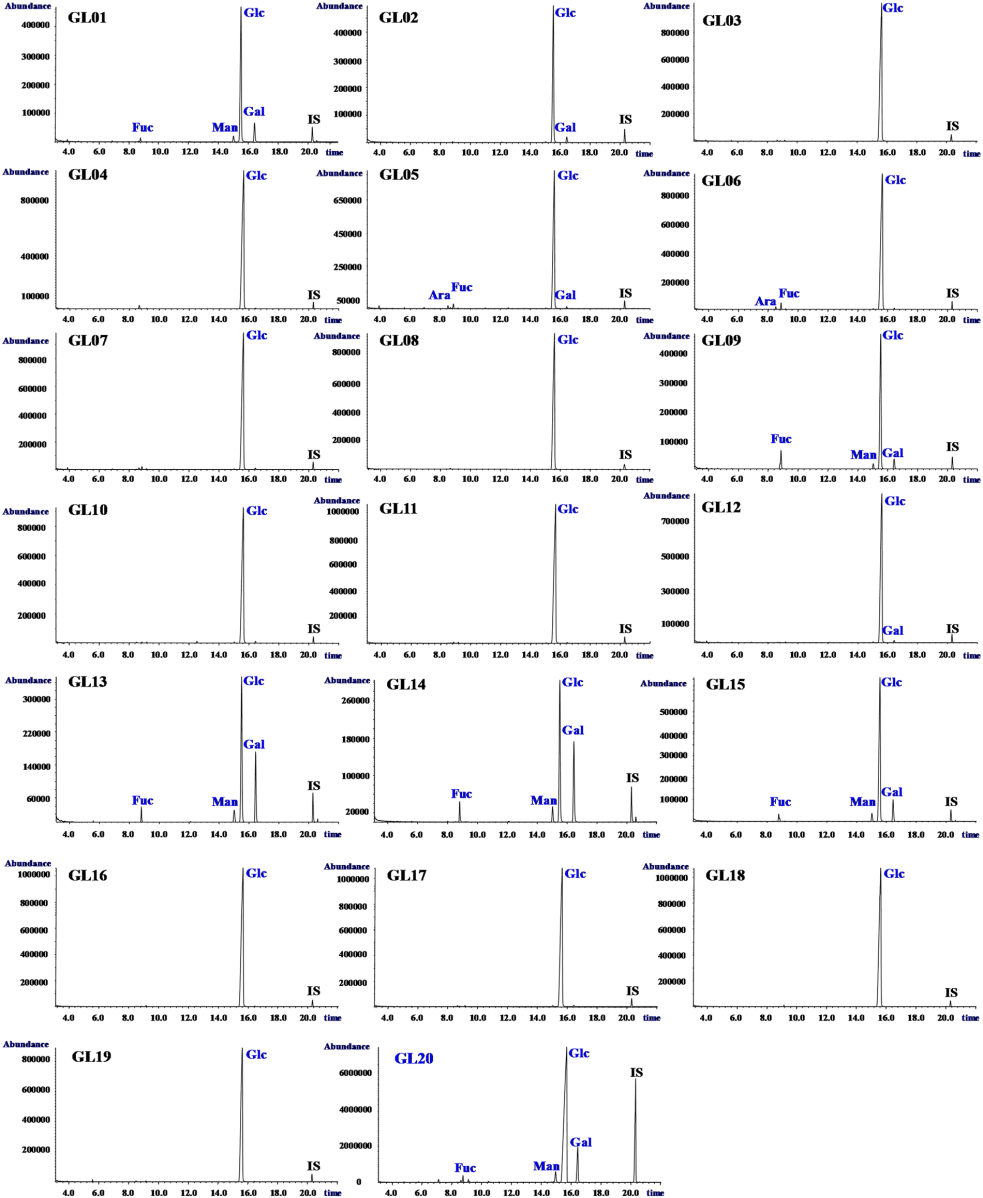
GC-MS profiles of methylated hydrolysates of polysaccharides from *G*. *lucidum* dietary supplements. **Ara**, arabinose; **Fuc**, fucose; **Man**, mannose; **Glc**, Glucose; **Gal**, Galactose; **IS**, internal standard. Sample codes were the same as in Table 1.

Fingerprints based on compositional monosaccharides have been successfully performed for the quality control of polysaccharides from *Ganoderma* ^13–15^, which demonstrated that compositional monosaccharides in fruiting body of *G*. *lucidum* collected from different regions of China were similar. Indeed, GC-MS is an excellent technique for the analysis of monosaccharides, which has several advantages such as high resolution and high sensitivity^16^. Therefore, GC-MS fingerprints based on compositional monosaccharides were also used for the authentication of *G*. *lucidum* dietary supplements. Figure 5 showed GC-MS fingerprints of compositional monosaccharides of polysaccharides in *G*. *lucidum* dietary supplements. The results showed the compositional monosaccharides of polysaccharides in *G*. *lucidum* (GL20) were fucose (Fuc), mannose (Man), glucose (Glc), and galactose (Gal) with the molar ratios of 2.3: 4.6: 100.0: 12.2, which were in accordance with the previous study^17^. Indeed, GC-MS analysis showed the types of compositional monosaccharides of polysaccharides in *G*. *lucidum* dietary supplements including GL01, GL09, GL13, GL14, and GL15 were similar to those of GL20 (Fig. 5 and Table 1), which suggested that *G*. *lucidum* polysaccharides could be found in these samples. However, types of compositional monosaccharides of polysaccharides in other *G*. *lucidum* dietary supplements (namely, GL02 to GL08, GL10 to GL12, and GL16 to GL19) were obviously different from those of *G*. *lucidum* (Fig. 5 and Table 1), which indicated that *G*. *lucidum* polysaccharides were not detected in these tested products. Therefore, GC-MS results further supported that *G*. *lucidum* polysaccharides were only found in 5 out of 19 (26.3%) tested products, which were in accordance with the results from saccharide mapping based on PACE analysis.

**Table 1:** Compositional monosaccharides of polysaccharides in *G*. *lucidum* dietary supplements and their measured ingredients.

Codes	Lot.	Monosaccharides and molar ratios^a^					Label ingredients	Measured ingredients	
		Ara	Fuc	Man	Glc	Gal		Triterpenes	Polysaccharides
GL01	X005	—^b^	2.4	4.0	100.0	2.6	Fruiting body	+^c^/+^d^	+/+
GL02	Not clear	—	—	—	100.0	4.0	27% polysaccharides +6% triterpenes	+/+	+/−^e^
GL03	1501901	—	—	—	1.0	—	Water extract	−^c^/−	+/−
GL04	MD14352A	—	—	—	1.0	—	13.5% polysaccharide +6% triterpenes	+/+	+/−
GL05	J0203	1.4	2.1	—	100.0	1.1	Fruiting body	+/−	+/−
GL06	250913-GLS	1.1	2.5	—	100.0		Fruiting body	+/−	+/−
GL07	20027414	—	—	—	1.0	—	10% polysaccharide	−/−	+/−
GL08	17365/4	—	—	—	1.0	—	Fruiting body	+/−	+/−
GL09	REV-1.12	—	8.9	3.8	100.0	7.0	Water extract: ethanol extract = 2:1	+/+	+/+
GL10	250913-GLS	—	—	—	1.0	—	Fruiting body	+/−	+/−
GL11	142786	—	—	—	1.0	—	Fruiting body +10% polysaccharides	+/−	+/−
GL12	ZFPG11110-WE	—	—	—	100.0	1.1	Reishi mushroom extract	±^f^/+	±/−
GL13	QYZG11403-St	—	8.5	8.5	100.0	48.0	Reishi mushroom extract	±/+	±/+
GL14	QYZG11311-St	—	11.0	10.0	100.0	55.0	Reishi mushroom extract	±/+	±/+
GL15	XTYGI1309-St	—	3.1	4.6	100.0	11.0	Reishi mushroom extract	±/+	±/+
GL16	AMGSGI	—	—	—	1.0	—	Reishi mushroom water extract	−/−	+/−
GL17	FPHDGI-1114	—	—	—	1.0	—	Reishi mycelia	±/−	±/−
GL18	PRMUSHPW16150126	—	—	—	1.0	—	Fruiting body	+/−	+/−
GL19	411346	—	—	—	1.0	—	Reishi mushroom extract	±/−	±/−
GL20	–	—	2.3	4.6	100.0	12.2	Authenticated material	+/+	+/+

^a^The data was presented as average of two determinations, their relative average deviation was less than 4%; ^b^Not detected; ^c^labeled (+) or unlabeled (+) ingredients; ^d^detected (+) or un-detected (−); ^f^uncertain (±).

### Molecular weights and contents of polysaccharides in *G*. *lucidum* dietary supplements

**Figure 6:**
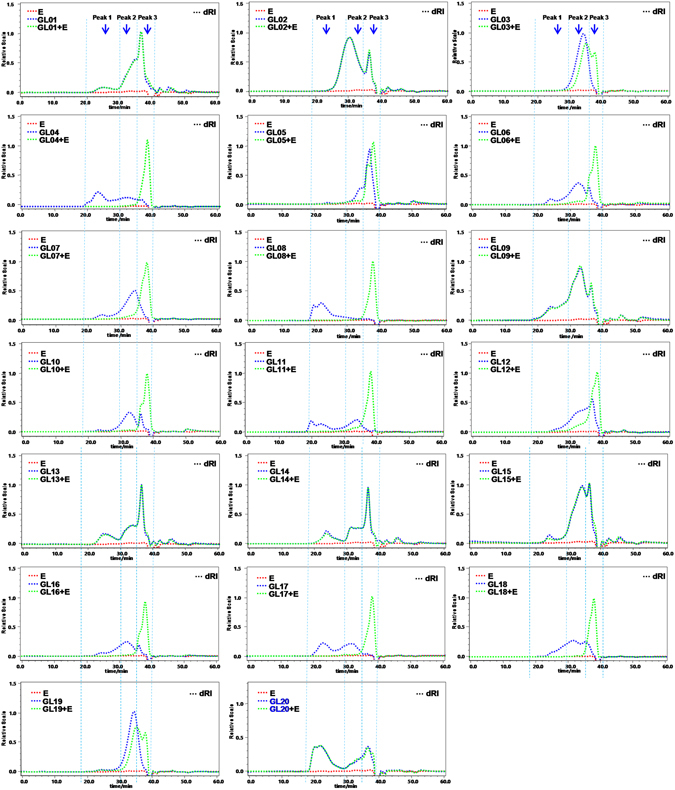
HPSEC chromatograms of polysaccharides in *G*. *lucidum* dietary supplements before and after α-amylase digestion. **E**, α-amylase; **GL** and **GL** **+** **E**, polysaccharides without and with α-amylase digestion, respectively. Sample codes were the same as in Table 1.

HPSEC-MALLS-RID based on *dn/dc* method has been proven as a powerful and efficient technique for the determination of contents and molecular weights of polysaccharides and their fractions from herbal medicines^18^. Indeed, HPSEC chromatograms and molecular weights of polysaccharides in *G*. *lucidum* collected from different regions of China were similar^15^. Therefore, polysaccharides from one batch of *G*. *lucidum* could be used as reference standards. In order to exclude the interference from the presence of additives such as starch or malto-dextrin, polysaccharides were treated with α-amylase before HPSEC-MALLS-RID analysis based on *dn/dc*. Figure 6 showed the HPSEC chromatograms of polysaccharides from tested products before and after α-amylase digestion. The results confirmed that α-amylase could digest all polysaccharides into small sugars in most of tested samples. However, the tested samples of GL01, GL02, GL09, GL13, GL14, and GL15 had negative response to α-amylase, which were in accordance with the results from colorimetric assay with I_2_-KI reagent. Indeed, HPSEC chromatograms of polysaccharides in GL01, GL09, GL13, GL14, and GL15 were similar to that of *G*. *lucidum* (GL20) (Fig. 6). Due to the relatively poor resolution of SEC and co-elution of various small molecules, *M*_*w*_ of peak 3 could not be accurately measured. Actually, peak 3 should be almost α-amylase hydrolysates of starch-like polysaccharides. Therefore, the molecular weights and contents of different fractions (peaks 1 and 2) were determined and calculated. The data showed that the molecular weights of polysaccharide in *G*. *lucidum* (GL20) were 1.052 × 10^6^ Da (peak 1) and 5.04 × 10^4^ Da (peak 2), respectively. Indeed, most anti-tumor β-glucans reported in *Ganoderma* contained the fraction with an average molecular weight of about 1.0 × 10^6^ Da^7^. Therefore, peak 1 could be considered as a quality marker in *G*. *lucidum*, which could be found in tested samples of GL01, GL09, GL13, GL14, and GL15. Their varied molecular weights might be attributed to different preparation processes of manufacturers. However, although peak 1 was also found in tested samples of GL02, GL03, GL12, and GL19, their compositional monosaccharides and PACE fingerprints of 1,3-β-D-glucanase hydrolysates were significantly different from those of *G*. *lucidum* (Table 2).

**Table 2:** Molecular weights and contents of polysaccharides in *G*. *lucidum* dietary supplements after α-amylase digestion.

Code	Peak 1			Peak 2			Total content (%)
	*Mw × *10^3^ kDa	*Mw/Mn*	Content^a^ (%)	*Mw* × 10^2^ kDa	*Mw/Mn*	Content (%)	
GL01	0.232	1.4	1.39	0.218	1.2	5.56	6.95
GL02	0.096	1.3	4.42	0.303	1.1	15.88	20.30
GL03	2.740	2.5	0.62	0.256	1.8	13.10	13.72
GL04	ND^b^	ND	0	0.725	1.2	1.48	1.48
GL05	ND	ND	0	0.950	1.4	0.70	0.70
GL06	ND	ND	0	1.139	1.3	0.56	0.56
GL07	ND	ND	0	1.201	1.3	1.33	1.33
GL08	ND	ND	0	0.922	1.1	0.12	0.12
GL09	1.872	2.1	0.93	1.551	1.1	1.88	2.81
GL10	ND	ND	0	0.699	1.1	0.50	0.50
GL11	ND	ND	0	1.051	1.6	0.69	0.69
GL12	0.581	2.2	1.09	0.476	1.1	6.67	7.84
GL13	0.196	1.3	1.21	0.257	1.1	1.84	3.05
GL14	0.502	1.4	1.40	0.388	1.1	2.09	3.49
GL15	1.375	1.2	0.70	0.654	1.5	7.58	8.28
GL16	ND	ND	0	0.407	1.4	1.20	1.20
GL17	ND	ND	0	0.291	1.5	0.29	0.29
GL18	ND	ND	0	0.347	2.4	0.72	0.72
GL19	2.297	1.6	0.55	0.228	1.9	13.04	13.59
GL20	1.052	1.8	1.61	0.504	1.3	0.54	2.15

^a^Content of polysaccharides in the raw material, all data were the average of two measurements with coefficient of variation <5%; ^b^Not detected in the sample; Peaks were the same as in Fig. S4; Sample codes were the same as in Table 1.

In summary, triterpenes and polysaccharides are officially considered as major active components in *G*. *lucidum*. Therefore, it is very important to determine whether the products contain triterpenes and polysaccharides derived from *G*. *lucidum*. Due to both triterpenes and polysaccharides can be found in *G*. *lucidum*, its products should, at least, have chemical characters of either triterpenes or polysaccharides based on their ethanol or water extract and their obvious polarity variations. Quality consistency of the dietary supplements could be considered as desirable if their detected ingredients were in accordance with their labels. Unfortunately, the measured ingredients, only in 5 out of 19 (26.3%) tested samples, were in accordance with their labels according to the results mentioned above (Table 1), which was similar to the data of DNA barcoding test^5^. Although only 19 batches of products were collected and analyzed, they almost represented the *G*. *lucidum* dietary supplements available in USA market. Actually, ten batches are enough for evaluating the quality consistency of tested sample (China Pharmopoeia 2015). Therefore, this study suggested the quality consistency of *G*. *lucidum* dietary supplements collected in USA was extremely poor, which should be carefully investigated. Furthermore, the results also suggested that saccharide mapping based on PACE analysis, and HPSEC-MALLS-RID based on *dn/dc* had great potential for routine quality evaluation of polysaccharides from dietary supplements.

## Materials and Methods

### Materials and chemicals

Nineteen batches (GL01 to GL19) of *G*. *lucidum* dietary supplements were purchased, in February or March 2015, directly from general e-commerce sites such as Amazon.com and eBay Inc. in the United States, and one batch of authenticated fruiting body of *G*. *lucidum* (GL20) was collected from Shandong Province of China (Table 1). Identity of *G*. *lucidum* fruiting body was confirmed by Professor Xiaolan Mao, Institute of Microbiology, China Academy of Sciences. The voucher specimens were deposited at the Institute of Chinese Medical Sciences, University of Macau, Macao, China.

D-glucose, α-amylase, starch (ST), and acetic anhydride were purchased from Sigma (St. Louis, MO, USA). Laminaribiose (DP2), laminaritriose (DP3), and laminaritetraose (DP4), β-1,3-D-glucan (GN), and β-1,3-D-glucanase were purchased from Megazyme (Wicklow, Ireland), and 8-aminonaphthalene-1,3,6-trisulphonic acid (ANTS) was purchased from Tokyo Chemical Industry (Tokyo, Japan). Silica gel 60 F_254_ TLC plates were obtained from Merck (Merck, Darmstadt, Germany). Polyacrylamide containing acrylamide/*N*, *N*-methylenebisacrylamide (19:1, *w/w*) was obtained from Bio-Rad (Hercules, CA, USA). Deionized water was prepared by a Millipore Milli-Q Plus system (Millipore, Bedford, MA, USA). All the other reagents were of analytical grade.

### Preparation of triterpenes and polysaccharides

Powder of each sample (1.0 g) was immersed in 20.0 mL of ethanol and refluxed in a Syncore parallel reactor (Büchi, Flawil, Switzerland) for 30 min at 78 °C according to previous report^10^. Then the extract was centrifuged at 4,000 × g for 10 min (Allegra X-15R centrifuge; Beckman Coulter, Fullerton, CA, USA). The supernatant was evaporated to dryness under vacuum using rotary evaporator (Büchi, Flawil, Switzerland), and the residue was dissolved in 2.0 mL of methanol. After filtration through a 0.22 μm membrane filter, the extract was used for HPTLC analysis. Finally, ethanol extracted residues were dried under vacuum at 45 °C and further used for polysaccharides preparation.

Microwave assisted extraction was used for polysaccharides extraction according to a previously reported method with modification^12^. Briefly, dried ethanol extracted residues (~1.0 g) were suspended in 25.0 mL of deionized water and extracted with microwave assisted extraction (Multiwave 3000, Anton paar GmbH, Graz, Austria). The microwave irradiation program was performed at 900 W and 90 °C for 7 min. Then the extract was centrifuged, and the supernatant (~25.0 mL) was evaporated to about 10.0 mL under vacuum using rotary evaporator. Then three times of solution volume of ethanol (95%, *w/v*) were added for the precipitation of crude polysaccharides. After the solution was kept at 4 °C for 12 h, centrifugation (4,500 × g for 15 min) was performed. The supernatant was collected and the powder of the supernatant was obtained by freeze-drying.

### HPTLC analysis of triterpenes

Determination of triterpenes in *G*. *lucidum* and its dietary supplements was performed according to a previously reported method with minor modification^10^. Briefly, all samples (5 μL) were applied on a 20 × 10 cm silica TLC plate with an AS30 HPTLC Applicator (Desaga GmbH, Germany). The bands, at 10 mm from the bottom edge, were 8 mm wide and 13 mm between two bands. The plate was developed to a distance of 90 mm with dichloromethane/ethyl acetate/petroleum ether/formic acid/ethanol, 8:3:9:0.8:0.5 (v/v/v/v/v) as mobile phase at room temperature. Finally, the developed plates were colorized with 10% (*v/v*) H_2_SO_4_ in ethanol, and heated at 110 °C for 10 min on a YOKO-XR plate heater (Wuhan YOKO technology Ltd., China). Then the plate was covered with transparent glass and photographed under white light and UV 365 nm, respectively.

### Analysis of polysaccharides

#### Colorimetric assay with I_2_-KI reagent

All samples (5 mg/mL, 200 μL) were mixed with 20 μL of I_2_-KI reagent, and then photographed under white light. Water and soluble starch were used as negative and positive control, respectively. Furthermore, all samples (5 mg/mL, 200 μL) were treated with α-amylase at a final concentration of 20 U/mL, and digested overnight (12 h) at 40 °C. Then, colorimetric assay was also performed.

#### Saccharide mapping based on PACE analysis

Saccharide mapping based on PACE analysis was performed according to the previous method with minor modification^19^. Briefly, polysaccharides of each sample (30.0 mg) were dissolved in 5.0 mL of hot water (60 °C). Then the compounds with molecular weights less than 3 kDa were removed by centrifugation (4000 × g, 25 min) with an ultra centrifugal filter (molecular weight cutoff: 3 kDa, Millipore, Billerica, MA, USA) for seven times.

The polysaccharide solution (1.0 mL) of each sample was mixed with α-amylase and 1,3-β-D-glucanase (the final concentration of 20.0 U/mL and 2.0 U/mL, respectively), and digested overnight (12 h) at 40 °C. The hydrolysates were dried, and then used for the derivatization with ANTS for PACE analysis. Polysaccharide solutions without enzymatic digestion were used as blank control after the treatment as described above. Reference polysaccharides including 1,3-β-D-glucan (5 mg/mL, 100 μL) and starch (5 mg/mL, 100 μL) treated with the corresponding enzymes, respectively, were used as positive control.

All samples (1–3 μL) were separated using a vertical slab gel electrophoresis apparatus, Mini-Protean Tetra System (Bio-Rad, Hercules, CA, USA). Electrophoresis on 30% (w/v) polyacrylamide as the resolving gel with a stacking gel of 8% (w/v) polyacrylamide was used for the separation of enzymatic hydrolytes. Samples were electrophoresed firstly at 200 V for 10 min and then at 700 V for 45 min. All runs were performed at least two times. Gels were imaged using an InGenius LHR CCD camera system (Syngene, Cambridge, UK) under UV 365 nm.

### GC-MS analysis

Compositional monosaccharides of polysaccharides in *G*. *lucidum* fruiting dietary supplements were investigated by using GC-MS analysis according to previous report with minor modification^20, 21^. Briefly, the sample (3.0 mg) was hydrolyzed with 2.0 M TFA (1.0 mL) at 95 °C in a sealed tube for 10 h. Then the hydrolysates were washed with methanol and evaporated to dryness before derivation with hydroxylamine hydrochloride and acetic anhydride at 90 °C for 30 min. Furthermore, the derivatives of mixed monosaccharide standards (1.0 mg/mL of Ara, Fuc, Gal, Glc, and Man, respectively) were prepared as described above. The derivatives were analyzed by using an Agilent 6890 gas chromatography instrument coupled to an Agilent 5973 mass spectrometer (Agilent Technologies, Palo Alto, CA). A capillary column (30 m × 0.25 mm, i.d.) coated with 0.25 μm film 5% phenyl methyl siloxane was used for separation. High purity helium was used as carried gas with a flow rate of 1.0 mL/min. The column temperature was set at 165 °C and held for 7 min for injection, then programmed at 5 °C/min to 185 °C and held for 5 min, then at 4 °C/min to 200 °C, and finally at 20 °C/min to 280 °C, and held for 2 min.

### HPSEC-MALLS-RID based on the dn/dc analysis

Contents and molecular weights of polysaccharides and their fractions in *G*. *lucidum* dietary supplements were simultaneously determined using HPSEC-MALLS-RID based on *dn/dc* method according to our previous report with minor modification^18^. Firstly, polysaccharide solutions (~5.0 mg/mL) of *G*. *lucidum* dietary supplement were treated with α-amylase at a final concentration of 20.0 U/mL in a total volume of 1.0 mL for 24 h at 40 °C to completely hydrolyzed additives (such as soluble starch, dextrin, and pullulan) into small sugars. Polysaccharide solutions without α-amylase, and α-amylase without polysaccharide solutions treated as described above, were used as blank control. Secondly, HPSEC-MALLS-RID measurements were carried out on a MALLS (DAWN HELEOS, Wyatt Technology Co., Santa Barbara, CA, USA) with an Agilent 1100 series LC/DAD system (Agilent Technologies, Palo Alto, CA, USA) equipped with columns of TSK-Gel G5000PW_XL_ (300 mm × 7.8 mm, i.d.) and TSK-Gel G3000PW_XL_ (300 mm × 7.8 mm, i.d.) in series at 35 °C. The MALLS instrument was equipped with a He-Ne laser (λ = 658 nm). An Optilab rEX refractometer (DAWN EOS, Wyatt Technology Co., Santa Barbara, CA, USA) was simultaneously connected. The *M*_w_ was calculated by the Zimm method of static light scattering based on the basic light scattering equation is as follows^22, 23^,

1 $$\frac{Kc}{{R}_{\theta }}=\frac{1}{{M}_{w}}(1+\frac{16{\pi }^{2}{\langle {S}^{2}\rangle }_{z}}{3{\lambda }^{2}}{\sin }^{2}(\frac{\theta }{2}))+2{A}_{2}C+\ldots $$

where *K* was an optical constant equal to [4*π* ^2^ *n* ^2^(*dn*/*dc*)^2^]/(*N*_*A*_ *λ* ^4^); *C*, polysaccharide concentration; *R*_*θ*_, Rayleigh ratio; *λ*, wavelength; *n*, refractive index of the solvent (0.9% NaCl aqueous solution); *dn*/*dc*, refractive index increment of polysaccharides in 0.9% NaCl aqueous solution, which is recommended as 0.15 mL/g according to our previous study^24^; *N*_*A*_, Avogadro’s number; *A*_2_, second virial coefficient.

Contents of polysaccharides were calculated based on the refractive index difference with *dn/dc* value according to the following equation^25^,

2 $${C}_{i}=\frac{\alpha ({V}_{i}-{V}_{i,baseline})}{{dn}/{dc}}$$

where *C*_*i*_ is the concentration of polymers; *α* is the RID calibration constant (in RI units per volt); *V*_*i*_ and *V*_*i*, *baseline*_ are the RID voltages of sample and baseline, respectively; *dn/dc* is the specific refractive index increment.

### Data analysis

The digital scanning chromatograms of PACE fingerprints were generated using Quantity-One software (version 4.6.2, Bio-Rad, Hercules, USA), and hierarchical cluster analysis of PACE fingerprints was also analyzed using Quantity-One with average linkage method.
